# Physiological Synchrony Across Modalities Within and Between Individuals: A Methodological Exploration of Crossmodality

**DOI:** 10.1111/psyp.70424

**Published:** 2026-10-19

**Authors:** Wolfgang Tschacher, Anu Tourunen, Petra Nyman‐Salonen, Virpi‐Liisa Kykyri

**Affiliations:** ^1^ University Hospital of Psychiatry and Psychotherapy University of Bern Bern Switzerland; ^2^ Department of Psychology, Faculty of Education and Psychology University of Jyväskylä Jyväskylä Finland; ^3^ Department of Psychology Faculty of Medicine, University of Helsinki Helsinki Finland

## Abstract

This methodological study aims at exploring several extensions of physiological synchrony. As yet, synchrony is commonly analyzed within the same physiological modality and between different people, whereas in the present project we were specifically interested in crossmodal synchronies between people (interpersonal), as well as within the same person (intrapersonal). In recent years, interpersonal synchrony has received increased attention as a research field and a methodology in various disciplines where social interaction is pivotal. Synchrony is often computed on a time scale of a few seconds, predominantly based on physiological measures or motor behavior. Systematic reviews and meta‐analyses have shown that interpersonal synchrony is a phenomenon that characterizes people's social interaction in different contexts. Synchrony arises spontaneously in social systems, often outside the awareness of the persons interacting, and is often considered a signature of relationship quality. Synchrony was also shown to be induced by salient stimulation contexts, for example by music synchronizing concert audiences. To explore such synchronies, we used a large dataset of 703 participants who listened to classical music in a concert setting. Heart rate, heart‐rate variability, electrodermal activity, breathing behavior, and respiration rate were recorded simultaneously in all participants. Using the surrogate synchrony algorithm (R package SUSY), which is based on cross‐correlations, the participants' crossmodal synchronies were determined for all 10 combinations of these 5 modalities. The psychological meaning of physiological synchronies is the goal of empirical aesthetics, and findings were published previously. The present study, however, concerns methodological topics of multimodal synchrony: do crossmodal synchronies arise intra‐ and interpersonally? Using surrogate tests as a control condition, we found that about half of such synchronies were significantly present in the dataset at substantial effect sizes. We additionally explored the lag structure of the underlying cross‐correlations to describe the leading‐following dynamics between synchronized physiological modalities. We expected that crossmodal synchronies of modalities representing sympathetic activation would be significantly synchronized positively (in‐phase synchrony). This was, however, not always the case, as co‐activation of sympathetic and parasympathetic signals was also found. In conclusion, the present findings on crossmodal synchronies provide novel information and suggest new measures for synchrony research, which is relevant for both induced synchrony and synchrony of social interaction.

## Impact Statement

Interpersonal synchrony is an increasing research field in social science disciplines. Synchrony arises spontaneously in social contexts and was also shown to be induced by salient stimulation contexts, for example, music. Both categories of synchrony, spontaneous and induced, can be assessed by the same statistical methodology: here windowed cross‐correlations with surrogate tests. Up to now, synchrony was exclusively analyzed within a single physiological modality. Here we were specifically interested in crossmodal synchronies, for example, the synchrony of one person's electrodermal activity and another person's heart rate, or the synchrony of electrodermal activity and heart rate within the same person. Such crossmodal synchronies constitute an entirely novel approach in the synchrony field. Crossmodal synchronies provide information relevant to various contexts including social interaction synchrony as well as induced synchrony.

## 1 Introduction

The present study has the goal to elaborate and extend the methodology of analyzing physiological synchronies. Current synchrony research is focused almost exclusively on synchronies within the same physiological modality and between people. Most measures are thus intramodal and interpersonal; we wished to complement this research by also exploring whether crossmodal and intrapersonal measures can be assessed. This is therefore an exploratory methodological study that aims to lay the foundations for ensuing psychological research.

In recent years, interpersonal synchrony has received much attention as a research field in disciplines where social interaction is pivotal, such as clinical psychology and psychotherapy research, social psychology, developmental psychology and a number of further social sciences. Synchrony is commonly defined as the coordination (or coupling, entrainment, attunement, contagion, …) between individuals who share the same stimulus environment or are engaged in communicative acts. There is an increasing literature on such interpersonal synchrony based on time series of body movement (e.g., Georgescu et al. 2020; Coeugnet et al. 2025), of sympathetic arousal (Karvonen et al. 2016; Tschacher et al. 2025) and further physiological variables (Tschacher and Meier 2020; Coutinho et al. 2021). Systematic reviews and meta‐analyses have shown that interpersonal synchrony is a phenomenon that characterizes people's social interaction in various contexts (Atzil‐Slonim et al. 2023; Gregorini et al. 2025; Kleinbub 2017). As a rule, synchrony arises spontaneously in sociality, often outside the awareness of the persons interacting. Synchrony research is inherently temporal because it examines the dynamics of measured variables over time, typically represented as time series.

In addition to such coordinated behavior and physiological activation in social interaction, synchrony has also been studied as *induced by the environment*. The field of ‘coordination dynamics’ (Haken et al. 1985; Kelso 2003) modeled synchronized behavior as a signature of self‐organized dynamics, using instructions to synchronize behavior or *zeitgebers* such as metronomes. Synchrony can also be induced implicitly by external stimuli such as music, movies, or rituals (Stuldreher et al. 2023; Konvalinka et al. 2011). There is ample evidence that music listeners become significantly synchronized in concert settings, where synchrony of listeners' physiological signals is found (Tschacher, Tröndle, and Weining 2024). Such synchrony arises on a phasic time scale of few seconds. Listeners' self‐reported aesthetic experiences, emotions, and personality traits were linked with the extent of physiological synchronies induced by the music; only synchronies within the same modality were considered (i.e., intramodal synchronies). Listeners showed significant intramodal interpersonal synchronies of heart rate, heart rate variability, respiration rate and electrodermal activity, yet not of respiration behavior (which represents the oscillatory time series of inhalation and exhalation). These synchrony findings are consistent with research showing that musical stimuli can arouse and also relax listeners, in other words that music has an effect on sympathetic and parasympathetic activations of listeners' autonomic nervous systems (Lynar et al. 2017; Ellis and Thayer 2010; Wright et al. 2022). This activation is commonly experienced by listeners as ‘being moved’ emotionally (Menninghaus et al. 2015; Mori and Iwanaga 2017) and is linked with aesthetic experiences (Zickfeld et al. 2020). Such activation generates interpersonal synchrony as listeners tend to show elevated autonomic arousal predominantly at the same time in the course of a music piece as they go along with the music and its changing rhythm, loudness, frequency and melodic properties. In the present study, we observed induced synchrony that was exclusively caused by the musical stimuli; participants did not interact socially.

The goal of the present study was methodological—going beyond studying synchrony within the same modality, and illuminating the *cross‐modality* of physiological synchronies to explore candidate measures for later research, for example the synchrony between heart rate and electrodermal activity. Prior to assessing the psychological associations of crossmodal synchronies, the feasibility of computing these measures must be established in the first place. To our knowledge, this has not been done before using the longitudinal approach and timescale in the range of seconds implemented by synchrony research.

The timeseries needed to compute these synchronies were recorded in participants exposed to a chamber music piece by Ludwig van Beethoven. The large available sample of participants of a project targeting responses to live concert music (Tröndle et al. 2026) allowed assessing such *crossmodal* synchronies within the same person (i.e., intrapersonally) and between persons listening to the music at the same time (i.e., interpersonally). Additionally, we measured *intramodal* synchronies between different persons, hence intramodal interpersonal synchronies. The key novelty is thus that we examined both within‐person and between‐person synchrony across multiple physiological modalities in a large sample, in which synchronies were induced by the shared external stimulus (music) and without interaction or speech as potential confounds (a classical concert setting).

Owing to the methodological novelty of computing crossmodal synchronies, the present project is clearly exploratory. We concurrently measured five physiological modalities: heart‐rate (HR), heart‐rate variability (HRV), electrodermal activity (EDA), respiration behavior (RESP), and respiration‐rate (RR). The literature shows that music elicits physiological variables similarly in many listeners; thus, we expected that physiological signals representing sympathetic arousal should show the same polarity of their variability over time, thus significant positive correlations presenting as in‐phase synchrony. Anti‐phase synchrony should emerge between a physiological signal indicating parasympathetic activation and another signal indicating sympathetic activation (research question RQ1). RQ1 applies to six out of ten intrapersonal synchronies since respiration behavior RESP is not a signature of either sympathetic or parasympathetic activation (cf. Table 1 for an overview). RQ1 also applies to 6 out of 15 interpersonal synchronies for the same reason of excluded respiration behavior and additionally because intramodal interpersonal synchronies are irrelevant to RQ1. Second, we expected that findings might be consistent for crossmodal synchronies in both the intra‐ and interpersonal condition (research question RQ2). RQ2 applies to all 10 crossmodal synchronies.

**TABLE 1:** Three types of synchronies were computed: Crossmodal intrapersonal, crossmodal interpersonal, intramodal interpersonal. *n*: Sample sizes prior to consideration of missing data.

	Intramodal (*n* = 5 modalities)	Crossmodal (*n* = 10 crossmodalities)
Intrapersonal (*n* = 703 persons)	**–** (no synchrony possible of the same modality within the same person)	*Crossmodal intrapersonal synchrony*: synchronies between different modalities within persons (up to *n* = 703 per crossmodality combination)
Interpersonal (*n* = 23,213 interpersonal permutations)	*Intramodal interpersonal synchrony*: synchronies of the same modality between persons (up to *n* = 23,213 per modality)	*Crossmodal interpersonal synchrony*: synchronies between different modalities between persons (up to *n* = 46,426 per crossmodality combination)

The synchrony computations used in this study enable testing which modality in the crossmodal synchronies has a leading function (research question RQ3). The leading vs. following research question is fully exploratory as no empirical evidence exists to date in synchrony research. As yet, leader‐follower dynamics have exclusively been studied in the same modality (intramodally) and in interaction contexts (e.g., Konvalinka et al. 2010; Kupper et al. 2015; daSilva and Wood 2025). Crossmodal “leading” does not necessarily imply a causal relationship between modalities; research question RQ3 may merely indicate which physiological modality shows faster responses than the other modality it is linked with.

## 2 Methods

### 2.1 Music Stimuli

In a series of 11 concerts, the first movement of Ludwig van Beethoven's String Quintet Op. 104 in C minor was featured. In this study, participants were members of all audiences listening to this 10‐min segment. According to Kurth (2002), this composition is one of Beethoven's lesser‐known works and is one of only four string quintets Beethoven published. It is, in fact, an arrangement of his earlier Piano Trio, Op. 1, No. 3. The quintet version—scored for two violins, two violas, and a cello—was completed in 1817 and first performed in 1818. The idea for this arrangement originated from an enthusiastic amateur musician who initially presented his own version of the piece to Beethoven.

The first movement, Allegro con brio (lively and spirited), is characterized by intense and turbulent moods, with frequent tempo changes and a broad tonal range. The musical expression shifts from sorrowful, lyrical passages to joyful, dance‐like sections, and even to resolute, march‐like themes. Given these dynamic musical features, it is likely that listeners' emotional responses to the piece varied considerably. In the questionnaires after the respective concerts, the majority of participants gave the piece high ratings on the scale “I liked this piece” that ranged from 1 to 5 (median 5; mean rating 4.38, SD 0.81). Correspondingly, the majority of listeners rated the Beethoven low on the scale “I felt bored” (median 1; mean rating 1.75, SD 0.95). When a subgroup of participants was interviewed 6 weeks after the concert to reflect on their experiences, differing emotional reactions were found: While some reported feeling deeply moved by the music, others did not experience any particularly strong emotions (Böndel et al. 2025).

This context, audiences listening collectively to a piece of music, was chosen as the stimulus for the present investigation of crossmodality for a number of reasons. First, a large number of audience members were participants in a project on aesthetic experiences, the Experimental Concert Research project,^1^ which provided an opportunity to analyze physiological signals that were recorded simultaneously in people listening to the same music at the same time. Second, the Beethoven piece was always played first at the beginning of an evening concert, so that listeners' responses may be considered comparable, even when recordings were collected in the course of 11 concerts. Third, physiological interpersonal synchrony within the same modality (intramodal) was found to be significant in these audiences (Tschacher, Tröndle, and Weining 2024).

### 2.2 Physiology of the Autonomic Nervous System

The five physiological modalities heart‐rate (HR), heart‐rate variability (HRV), electrodermal activity (EDA), breathing behavior (RESP), and respiration‐rate (RR) provide different information about the bodily activation of a person. Let us briefly recall which information is represented by the various modalities (Omigie et al. 2021; Wright et al. 2022). The Autonomic Nervous System (ANS) consists of two branches. These ANS branches are anatomically and endocrinologically different and consist of the activating sympathetic system and the relaxing parasympathetic system. The peripheral signals that we measured in the present study are the result of sympathetic and/or parasympathetic influence (Pocock et al. 2013). The two cardiac signals HR (measured as beats per minute) and HRV are relatively convenient to record non‐intrusively. HR is modulated by both branches of the ANS, where higher HR values denote bodily activation and reduced relaxation. HRV is the subjectively imperceptible variability of the intervals between subsequent heart beats; higher values predominantly reflect parasympathetic activation (Berntson et al. 2007). Electrodermal activity is the activity of the sweat glands in the skin, which are innervated exclusively by the sympathetic system. The fast component of this activity, skin conductance response (SCR), represents the phasic dynamics of the sympathetic system and was used in the present study. Respiration provided two signals for this research: the time series of respiratory behavior as such (RESP) indicate at which moment a person inhales and exhales; hence, it provides an oscillatory variable that has complex relationships with the other variables and no linear association with the two ANS branches. RR, like HR, represents both branches of the ANS; high RR means arousal and activation, low RR is linked with relaxed states. RR and RESP are the two physiological modalities that in addition to reflecting ANS activity also underlie volitional control.

### 2.3 Recording of Physiological Time Series

The data obtained are physiological time series measured in participants who were listening to the chamber music by Ludwig van Beethoven. The music was played as the first piece in all 11 live concerts. Three concerts were played by the Yubal Ensemble, eight by the Ensemble Epitaph. Concerts were organized by the research project “ECR—Experimental Concert Research” in April and May of 2022 in the two venues Pierre Boulez Saal and Radialsystem in Berlin, Germany.

All concerts were open to the public, and the audiences were informed about the possibility to participate in the ECR study. All adult participants who signed the written consent form were recruited, and the procedure was approved by the ethics committee of the Max‐Planck Society. The consent informed that participation was not possible under the influence of alcoholic beverages or psychoactive substances. About 75% of the concert audiences consisted of study participants. The present analyses are based on 703 participants with mean age 44.1 year (SD = 17.2 year). A total of 57.3% of participants were female. About 80% of participants had a university degree. Owing to Covid restrictions at the time of recording the data, participants were seated at a distance of at least 1 m from each other.

The present choice of physiological variables was guided by the necessity to accommodate to measurement under field conditions. Participants were equipped with sensors integrated in a glove to collect electrodermal and cardiac activity, and carried a strain‐sensitive belt placed over clothing beneath the thorax. Devices were manufactured by biosignalsplux (PLUX Wireless Biosignals, S.A.). The devices acquired the physiological data at a 1000‐Hz sampling rate. Blood‐volume pulse was captured by a photo‐plethysmographic sensor placed over one fingertip that measured the blood perfusion in the finger capillaries. Both heart‐rate (HR) and heart‐rate variability (HRV) were obtained from this blood‐volume pulse. HRV was computed by the RMSSD (root mean square of successive differences) procedure. Breathing behavior (RESP) and respiration‐rate (RR) were derived from belt distension. Electrodermal activity (EDA) was measured from electrodes attached to the middle phalanges of the middle and ring finger of the non‐dominant hand.

To analyze crossmodal synchrony, the five modalities (HR, HRV, EDA, RESP, RR) were preprocessed so that they had the same sampling rate. All five modalities underwent specific kinds of preprocessing.

#### 2.3.1 Cardiac Signals

The raw blood‐volume pulse data were processed using the BioSPPy library written in Python (Carreiras et al. 2015; see links in references) to extract the cardiac signals from the raw data of the devices. HR and HRV were computed based on the blood‐volume pulse, from which systolic peaks were detected to obtain inter‐beat intervals (IBI, the interval between adjacent systolic peaks). A filter was added to remove any IBIs shorter than 300 ms or longer than 2 s, and IBIs deviating by more than 20% from adjacent IBIs, all of which would indicate artifacts due to misidentified or missed peaks. After artifact removal, IBIs were converted to continuous heart‐rate (HR) at 1 Hz by linear interpolation, since IBIs may extend over 1 s. HR was expressed as beats‐per‐minute units.

HRV is a signal that is commonly sampled over periods much longer than each second. Therefore, additional preprocessing was necessary to arrive at 1‐Hz time series of HRV. All IBIs in a 30‐s moving window were computed and their RMSSD (root mean square of successive differences) was estimated. RMSSD specifically addresses the high‐frequency component of predominantly vagally mediated HRV, thus short‐term variability of this modality. The fitting of the 30‐s windows towards 1‐Hz sampling rate of the time series was achieved by moving the 30‐s windows in steps of 1 s, which provided an RMSSD estimate per each second.

#### 2.3.2 Electrodermal Activity

EDA was pre‐processed using Ledalab (Benedek and Kaernbach 2010) in the toolbox for biosignal processing BioSPPy. The continuous phasic component of the EDA signal was used in the analyses, as it reflects rapid sympathetic responses that may track ongoing changes in the music, while minimizing the influence of slower tonic drift and baseline shifts. The phasic signal was then down‐sampled to a 1‐Hz time series. The specific codes used for deriving event‐related responses are provided by links in the reference of Benedek and Kaernbach (2010).

#### 2.3.3 Respiratory Activity

Respiration behavior (RESP) depicts the oscillatory behavior of breathing with the locations in time of breathing in and out. Upon inhaling, the breathing belt is extended and the variable RESP increased. The raw data of the belt distention were low‐pass filtered (0.6 Hz), demeaned and down‐sampled to 1‐Hz time series. Respiration rate (RR) depended on determining the local peaks of RESP, analogous to HR, which depends on identifying IBIs. RR was derived from the RESP data, again using the BioSPPy library (Carreiras et al. 2015; code resp.py). Maximum peaks were located and RR was inferred from the intervals between peaks. RR was determined using a moving‐window approach to convert to the 1‐Hz sampling rate, in analogy to the estimation of HRV.

### 2.4 Computation of Synchrony

All time series were analyzed using the R package SUSY (*SU*rrogate *SY*nchrony Version V20_2; Tschacher 2023) to quantify the dyadic synchrony between different people (interpersonal synchrony) or the dyadic synchrony between two different modalities within the same person (crossmodal intrapersonal synchrony). Time series were available at 1‐Hz sampling rate. SUSY is based on cross‐correlations of the time series, and cross‐correlations were computed with time‐lags up to ±5 s. These correlations inform about simultaneous (lag = 0) as well as moderately time‐lagged synchronization of the physiological values analyzed (see Figure 1 for an example of crossmodal interpersonal synchrony). Thus, the amount of synchrony both of simultaneous as well as of activity with response delays of up to 5 s is contained in the aggregated cross‐correlations between time series.

**FIGURE 1:**
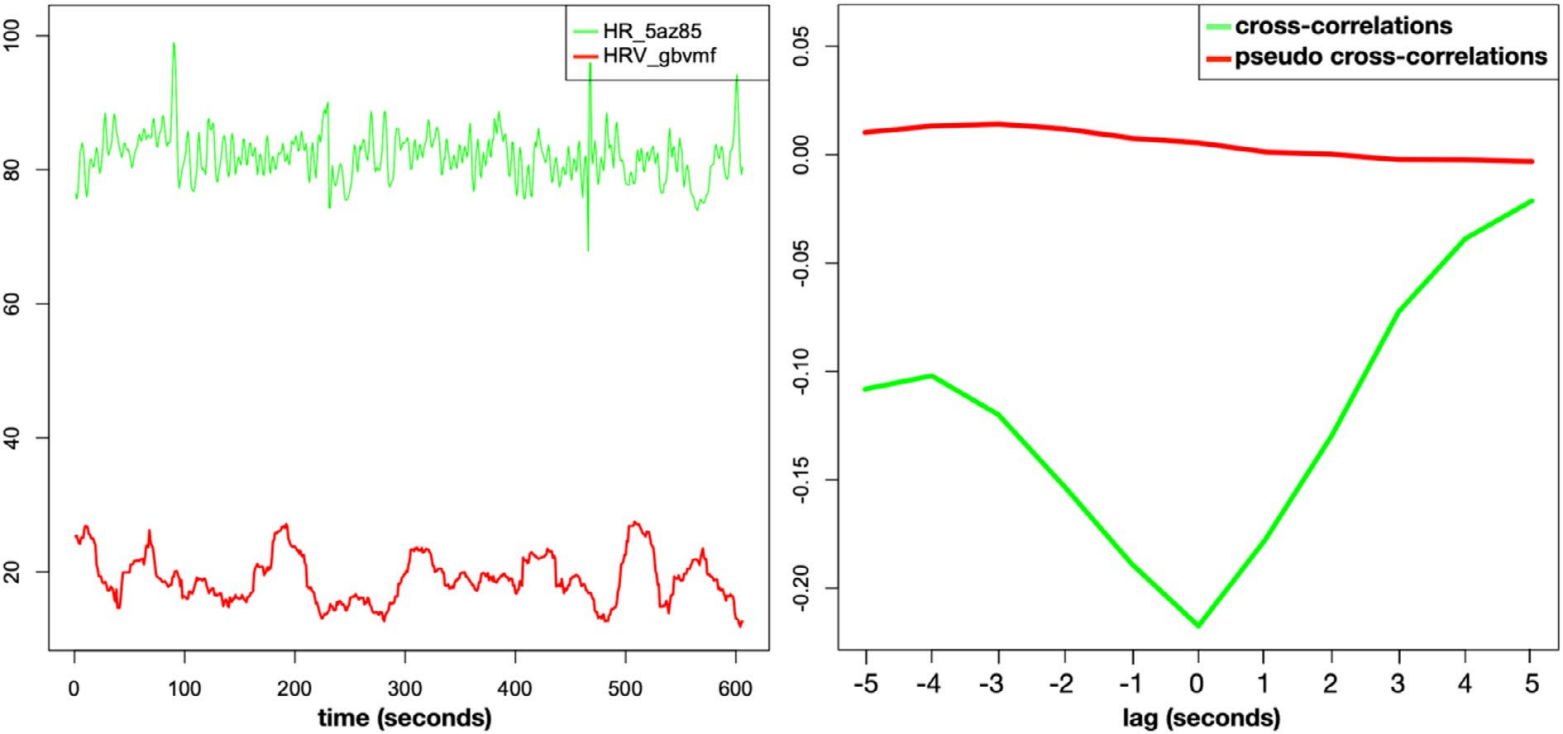
Example of a cross‐correlation function generated with surrogate synchrony (SUSY). Left: Time series of heart rate (participant code 5az85) and heart‐rate variability (participant code gbvmf); Right: Cross‐correlation function of these time series, which suggests anti‐phase synchrony.

To arrive at a control condition, surrogate tests are implemented in the SUSY algorithm: the time series received from participants listening to the music (the Beethoven piece had a duration of approximately 10 min, with small variations owing to the live performances) were divided into 30‐s non‐overlapping segments, and cross‐correlations were computed and aggregated across all lags in each segment, and then averaged over all segments of the whole music piece. In the surrogate tests, SUSY repeats this same procedure but with randomly shuffled segments, so that cross‐correlations are computed on data that did not actually refer to the same time, providing a value of ‘pseudo‐synchrony’. Such random shuffling is repeated many times, which creates a distribution of pseudo‐synchronies (in the 10‐min time series partitioned into 30‐s segments, 20 sections are available, thus 20 × 19 = 380 pseudo‐synchronies are generated). Then the real synchrony found during the music piece is contrasted against the pseudo‐synchronies. This contrast is expressed by an effect size measure of synchrony for each pair of time series. The effect size is computed as real synchrony minus mean of pseudo‐synchronies divided by the standard deviation of pseudo‐synchronies. We used these effect sizes, called ES_noabs_ in the output of SUSY; the value of this effect size expresses how much higher or lower the measured synchrony is than the mean pseudo‐synchronies, in units of standard deviations of pseudo‐synchronies. ES_noabs_ allows for so‐called anti‐phase synchrony (predominantly negative cross‐correlations as in the example of Figure 1, thus ES_noabs_ < 0), where more activity in one variable's time series is systematically linked with less activity in the other.

Cross‐correlations are a function of the lags between the time series. This cross‐correlation function need not have symmetrical shape: If variable A is the temporal ‘leader’ of activity in the synchronization dynamics, whereas variable B is ‘following’, this can be detected in the shape of the cross‐correlation function (cf. Figure 1), e.g., by higher cross‐correlation values when lags are negative than when they are positive. Thus, the lags of the cross‐correlations that in their sum form the overall measure of synchrony additionally allow for a differentiation between the synchrony component in which variable A assumes the leading role and variable B follows after. For instance, let the time series of variable A represent the heart‐rate of one person and B this same person's electrodermal activity; higher A‐B cross‐correlations at negative lags compared to positive lags of the cross‐correlation function would point to a leading role of A because negative lags correspond to heart‐rate changes occurring up to 5 s before electrodermal activity. Heart‐rate may thus have preceded, or may have triggered, electrodermal activity. Tests of the leading role were only performed in crossmodal synchronies. These tests would be meaningless in intramodal synchrony, for example, in EDA‐EDA synchrony.

The complete dataset comprised 703 participants, each of whom provided five modalities of physiology (HR, heart‐rate; HRV, heart‐rate variability; EDA, electrodermal activity; RESP, respiration behavior; RR, respiration‐rate). As detailed in the previous section, this sample size was not always reached owing to missing data. This sample therefore results in up to 703 different *crossmodal intrapersonal synchronies* in each of 10 different cross‐modality combinations (HR‐HRV, HR‐EDA, etc.).

Using the permutation option of the SUSY algorithm, *intramodal interpersonal synchronies* for each modality were also analyzed. Permutation was done separately in each of the 11 concerts because interpersonal synchrony is only meaningful between persons who attended the same concert. Concerts differed with respect to the number of participants, ranging between 85 and 38, and with respect to the duration of the Beethoven piece (as these were live performances), which lasted between 568 and 633 s. In a concert with 85 participants, 85 × 84/2 = 3570 permutations are possible. Based on the empirical sample sizes of each concert, up to *n* = 23,213 permutations were computed overall. This is the upper number of intramodal interpersonal synchronies.

The upper number of *crossmodal interpersonal synchronies* in each of the 10 crossmodality combinations is twice this number, hence *n* = 46,426. The reason for this, given in an example, is that the interpersonal combination personA and personB creates two crossmodal synchronies for two modalities, for example, HR and EDA: personA‐HR and personB‐EDA as well as personA‐EDA and personB‐HR. Table 1 illustrates the different kinds of synchronies that were computed as raw variables of overall synchronizations.

As a last step, to curb the large number of thousands of dyadic synchrony scores and reduce the hierarchical complexity of the data set, we aggregated these scores for each individual participant. This results in mean scores per participant that were called ‘synchrony contribution’ in several previous publications (Tschacher, Greenwood, Weining, et al. 2024; Tröndle et al. 2026).

### 2.5 Statistical Analyses

To assess which of the three types of synchronies (Table 1) were significantly present, we used one‐sample *t*‐tests for the respective mean ESnoabs of the three types against the null hypothesis that mean ESnoabs = 0. The results are provided in Table 2. Owing to the number of tests, with 10 crossmodalities in intrapersonal synchronies and 15 crossmodalities in interpersonal synchronies, we applied Bonferroni alpha adjustment on these analyses. For instance, the significant probability *p* of the 10 tests of crossmodal intrapersonal synchronies was changed to *p* = 0.05/10 = 0.005 and of the interpersonal synchronies to *p* = 0.05/15 = 0.0033. Prior to all one‐sample *t*‐tests, we conducted null models of the synchrony scores to analyze the variance that can be explained by a random effect ‘concert’. The respective Wald tests were insignificant throughout, which pointed to a minimal influence of the hierarchical level, thus of which concert the participants had attended. It was therefore not necessary to center the synchrony scores in ‘concert’.

The significance of leading synchronies was assessed by pairwise *t*‐tests between cross‐correlations of lags < 0 and those with lags > 0 (Table 4). For example, in the crossmodal synchronies EDA‐HR, lags < 0 denote EDA as the first variable, variable1 in the pair EDA‐HR, which is termed ‘ESnoabs(lead1)’ in Table 4. The pairwise *t*‐tests indicate whether the respective mean of ‘ESnoabs(lead1)’ or of ‘ESnoabs(lead2)’ is significantly larger, hence whether either EDA or HR was the leading variable. Again, alpha adjustment was applied.

## 3 Results

### 3.1 Synchronies

All three kinds of synchronies defined in Table 1 provided several significant crossmodal and intramodal synchronies in the interpersonal couplings and significant crossmodal synchronies in intrapersonal couplings.

The intramodal interpersonal synchronies (Table 2) constitute a replication of published results, where analyzing synchrony between people but within the same modality was the standard. Interpersonal synchronies of electrodermal activity (EDA), heart rate (HR), and respiration rate (RR) were highly significant and showed in‐phase couplings as in previous publications (cf. Table 1 in Tschacher, Greenwood, Weining, et al. 2024; the mean ES values reported there differ from the present Table 2 owing to different methods of aggregation and alpha correction). Respiration (RESP) was not interpersonally synchronized, as was expected and reported previously. The significant synchronies of Table 2 are considered as proof‐of‐principle that listeners' physiological activations resonate with the music stimulus the listeners were sharing (Tschacher, Greenwood, Ramakrishnan, et al. 2023; Tschacher, Greenwood, Egermann, et al. 2023; Tschacher, Greenwood, Weining, et al. 2024; Czepiel et al. 2021) and that synchrony computation is a reliable method to demonstrate this phenomenon.

**TABLE 2:** Intramodal interpersonal synchronies of audience participants.

Intramodal synchronies of variables EDA, HR, HRV, RESP, RR			
Synchrony of…	Interpersonal		
	Mean ESnoabs; *n*	95% CI	One‐sample *t* (test if ESnoabs ≠ 0)
EDA‐EDA	0.67; *n* = 657	[0.55, 0.79]	11.12*
HR‐HR	1.94; *n* = 674	[1.70, 2.18]	15.69*
HRV‐HRV	1.01; *n* = 669	[0.19, 1.82]	2.43
RESP‐RESP	−0.00; *n* = 687	[−0.01, 0.01]	−0.41
RR‐RR	1.92; *n* = 676	[1.45, 2.40]	7.95*

*Note:* Parameter settings in SUSY‐V20: maxlag 5 s; segment size 30 s; Beethoven piece duration ≈10 min. Bold print *: One‐sample *t*‐tests significant after Bonferroni‐correction (Bonferroni‐corrected *p*: 0.05/15 = 0.0033).

Abbreviation: CI, confidence interval.

We continue with results concerning crossmodal synchronies, which were available both for the intrapersonal and interpersonal condition (Table 3). Crossmodal interpersonal synchronies were insignificant in all four combinations with RESP, respiration behavior. This is also an expected finding because RESP‐RESP synchrony was not significantly coupled interpersonally—the shared music stimulus did not afford synchronized breathing in listeners (Table 2) across the duration of the Beethoven piece. Breathing not being systematically entrained by the music is consistent with the absence of breathing being coupled with other modalities, which themselves provided significant intramodal interpersonal synchronies.

We found four crossmodal interpersonal combinations that showed significant in‐phase synchronies: EDA‐HR, EDA‐HRV, EDA‐RR and HR‐RR. According to research question RQ1, the in‐phase polarity of synchronies EDA‐HR, EDA‐RR and HR‐RR is as expected, as is the anti‐phasic synchrony HR‐HRV (Laborde et al. 2022; Goessl et al. 2017). This was not the case with the synchrony of EDA‐HRV, where sympathetic arousal (higher EDA) was significantly linked with parasympathetic activation (higher HRV).

Crossmodal intrapersonal synchronies were found to be significantly in‐phase for EDA‐HR and EDA‐HRV. Both findings are consistent with expectations concerning RQ2 that crossmodal synchronies may be analogous in both the interpersonal and intrapersonal conditions. Whereas EDA‐HR synchrony is additionally in line with expectations (RQ1), EDA‐HRV synchrony again is not. Three further intrapersonal synchronies are of the anti‐phasic type: HR‐HRV, HRV‐RR and RESP‐RR. HR‐HRV synchrony is consistent with expectations related to RQ1 and RQ2, as sympathetic and parasympathetic signatures are negatively coupled (RQ1) and this is true in the respective inter‐ and intrapersonal condition (RQ2). HRV‐RR synchrony is as expected for RQ1 but not RQ2. The significant anti‐phasic RESP‐RR synchrony is an isolated finding in that it is the only indication of a significant coupling of respiration behavior with any other physiological signal and is only observed in the intrapersonal condition.

In overview of crossmodality tests listed in Table 3, among the interpersonal synchronies relevant to RQ1, four out of six tests were as expected, significantly inconsistent with expectations in one instance (EDA‐HRV) and insignificant in another (HRV‐RR). Intrapersonal synchronies were consistent with expectations in three out of six tests, significantly inconsistent in one instance (again, EDA‐HRV) and insignificant in two instances. RQ2 addressing the consistency of interpersonal with intrapersonal findings was assumed as consistent if both were significantly in‐phase or anti‐phase or both insignificant. RQ2 was as expected in six out of ten intermodal synchronies.

**TABLE 3:** Intrapersonal and interpersonal synchronies of audience participants.

Crossmodal synchronies of combinations of variables EDA, HR, HRV, RESP, RR								
Synchrony of…	Intrapersonal				Interpersonal			
	Mean ESnoabs	*n*	95% CI	One‐sample *t* (test if ESnoabs ≠ 0)	Mean ESnoabs	*n*	95% CI	One‐sample *t* (test if ESnoabs ≠ 0)
EDA‐HR	2.20	652	[1.52, 2.88]	6.37*	0.37	701	[0.28, 0.46]	7.77*
EDA‐HRV	6.75	647	[5.33, 8.16]	9.37*	0.64	701	[0.49, 0.79]	8.57*
EDA‐RESP	0.09	663	[0.01, 0.18]	2.08	−0.01	703	[−0.02, 0.00]	−1.16
EDA‐RR	−0.85	653	[−1.80, 0.10]	−1.75	0.28	702	[0.15, 0.41]	4.29*
HR‐HRV	−9.50	654	[−11.33, −7.68]	−10.22*	−0.83	689	[−1.04, −0.61]	−7.56*
HR‐RESP	0.14	680	[0.03, 0.25]	2.45	−0.00	703	[−0.01, 0.01]	−0.74
HR‐RR	−0.39	667	[−1.82, 1.04]	−0.54	1.69	703	[1.52, 1.87]	19.16*
HRV‐RESP	0.01	675	[−0.19, 0.21]	0.08	−0.02	703	[−0.04, −0.00]	−2.46
HRV‐RR	−3.07	664	[−4.88, −1.27]	−3.34*	−0.11	702	[−0.35, 0.14]	−0.86
RESP‐RR	−0.48	687	[−0.61, −0.35]	−7.21*	−0.01	698	[−0.02, 0.00]	−1.47

*Note:* Parameter settings in SUSY‐V20: maxlag 5 s; segment size 30 s; Beethoven piece duration ≈10 min. Bold print *: one‐sample *t*‐tests significant after Bonferroni‐correction (intrapersonal Bonferroni‐corrected *p*: 0.05/10 = 0.005; interpersonal Bonferroni‐corrected *p*: 0.05/15 = 0.0033).

Abbreviation: CI, confidence interval.

Table 4 lists the findings on the respective leading variable of crossmodal synchronies presented in Table 3. In the interpersonal condition, the four in‐phase synchronies EDA‐HR, EDA‐HRV, EDA‐RR, and HR‐RR that were significant in Table 3 pointed to significant differences between the synchronized variables, where the second variable of the respective pair was the leading variable. This was true for HR (followed by EDA), HRV (followed by EDA), and RR (followed by EDA and HR). The leading role was the same only in intrapersonal EDA‐HR synchrony, thus, as expected (RQ2) in one instance. The anti‐phase HR‐HRV of Table 3 showed higher anti‐phase activity for HR (followed by HRV) in Table 4. This leader‐follower relation for HR‐HRV was not significant in the intrapersonal condition, thus not in line with RQ2. The synchrony HRV‐RR, which was not significant in Table 3, pointed to a significant (anti‐phasic) leading role of RR, which is as expected based on RQ2.

In intrapersonal synchronies, the leader‐follower tests of the in‐phase synchronies of Table 3 for EDA‐HR and EDA‐HRV provided leading variable HR (followed by EDA) and EDA (followed by HRV). The anti‐phase synchronies of Table 3, HR‐HRV, HRV‐RR and RESP‐RR, showed insignificant leading in the HR‐HRV link, anti‐phasic RR leading (followed by HRV and RESP). Two synchronies were insignificant in Table 3 but significantly lagged in Table 4: in EDA‐RESP synchrony, EDA was the leading variable; in HR‐RESP synchrony, RESP was the leading variable.

**TABLE 4:** Intrapersonal and interpersonal synchronies of audience participants with pairwise *t*‐tests to indicate the leading variable. Only significant synchronies of Table 3 considered.

Crossmodal synchronies of combinations of variables EDA, HR, HRV, RESP, RR. Which is the leading variable?										
Synchrony of…	Intrapersonal					Interpersonal				
	Mean ESnoabs (lead1)	Mean ESnoabs (lead2)	*n*	95% CI of difference	Pairwise *t* test if lead2 > lead1	Mean ESnoabs (lead1)	Mean ESnoabs (lead2)	*n*	95% CI of difference	Pairwise *t* test if lead2 > lead1
EDA‐HR	−3.76	11.98	652	[13.2, 18.3]	12.24*	0.49	1.10	701	[0.39, 0.85]	5.24*
EDA‐HRV	14.30	9.27	647	[−8.37, −1.68]	−2.95*	0.86	1.46	701	[0.29, 0.95]	3.28*
EDA‐RESP	0.50	−0.05	663	[−0.92, −0.20]	−3.06*	0.00	−0.01	697	[−0.06, 0.02]	−1.05
EDA‐RR	0.56	−3.63	653	[−7.29, −1.10]	−2.66	−0.18	1.32	702	[1.18, 1.81]	9.43*
HR‐HRV	−21.01	−15.55	676	[−1.38, 12.3]	1.57	−2.14	−0.99	689	[0.63, 1.67]	4.36*
HR‐RESP	−2.10	4.03	680	[5.23, 7.04]	13.30*	0.02	0.00	697	[−0.07, 0.04]	−0.69
HR‐RR	−2.87	2.27	669	[1.46, 8.83]	2.74	2.29	4.12	703	[1.42, 2.24]	8.81*
HRV‐RESP	0.06	−0.23	675	[−1.14, 0.57]	−0.66	−0.04	0.03	697	[−0.00, 0.15]	1.94
HRV‐RR	13.82	−26.51	665	[−47.4, −33.2]	−11.18*	0.28	−1.01	697	[−1.87, −0.69]	−4.27*
RESP‐RR	−0.13	−1.11	687	[−1.56, −0.40]	−3.34*	−0.03	−0.01	697	[−0.05, 0.08]	0.53

*Note:* Parameters SUSY‐V20: maxlag 5 s; segment size 30 s; CI, confidence interval of mean differences lead2‐lead1; Beethoven piece duration ≈10 min. Reading example: *EDA‐HR synchrony*: lead1 is EDA; lead2 is HR. Bold print *: pair‐wise *t*‐tests significant after Bonferroni‐correction (Bonferroni‐corrected *p*: 0.05/10 = 0.005).

Espectations concerning RQ2 were fulfilled when the same leading variable was significantly found in the interpersonal and intrapersonal synchronies. This was the case in two out of five instances, in EDA‐HR and HRV‐RR synchronies; here, the leader‐follower relation was the same in interpersonal and intrapersonal synchronies.

Counting the numbers of significant leading roles among the five physiological variables showed that EDA, HR, HRV, RESP, RR differed: HR (3), EDA (2), HRV (1), RR (6), RESP (1). The followers were HR (2), EDA (5), HRV (4), RESP (2).

## 4 Discussion

We have explored the feasibility of computing *crossmodal* synchronies among different modalities of physiology within the individual person (intrapersonal synchronies) and between persons (interpersonal synchronies). The large sample of this study and the availability of five simultaneously recorded physiological modalities allowed us to examine both intra‐ and interpersonal synchrony within the same dataset. All interpersonal synchronies can have emerged in the participants exclusively because of the shared musical stimuli, as participants of concert audiences were resting in their seats at considerable physical distances; in accordance with the setting of a classical concert, they did not interact with one another. The goal of this study was methodological: we aimed to explore which types of synchrony were significantly present and are thus candidates for implementation in future synchrony research. In the second line, we wished to interpret findings on the basis of our theoretically derived research questions.

The present research approach opens a window into the crossmodal dynamical relations between different physiological signals at a time scale of a few seconds; in order to be significant, such relations needed to occur continuously, or at least frequently, during the music piece of about 10 min duration. Of five significant crossmodal interpersonal synchronies (Table 3, right panel), four concurred with the expectation of research question RQ1 (“sympathetic signals should be synchronized in‐phase with other sympathetic signals, and anti‐phase with parasympathetic signals”).

There is a qualitative difference between interpersonal and intrapersonal synchronies that must be heeded when interpreting the findings. Interpersonal synchronies represent exclusively the synchrony that is caused by the stimulus environment, whereas intrapersonal synchronies in addition may reflect physiological associations occurring within the person's body. This may help explain why our original expectation concerning research question RQ2 (“consistent synchronies in the intra‐ and interpersonal condition”) was not consistently met. For example, respiratory arrhythmia denotes the coupling of respiration behavior (RESP) and parasympathetic activity: when inhaling (the variable RESP is increased) parasympathetic activity is decreased and heart rate (HR) will increase. Respiratory arrhythmia is only valid *within* the person, but not interpersonally *between* persons, since breathing per se was not found to be synchronized (Table 2): the lack of synchronized breathing (RESP‐RESP) can explain the nonsignificance of all crossmodal interpersonal synchronies in which RESP was involved. The intrapersonal EDA‐RESP and HR‐RESP synchronies of Table 3, however, would have been largely consistent with respiratory arrhythmia (but unfortunately did not survive Bonferroni correction). Yet the leading analysis (Table 4) shows that, for intrapersonal HR‐RESP synchrony, breathing has a significant leading role with HR following, whereas the leading role in EDA‐RESP synchrony appears reversed.

One of the respiration‐linked crossmodal findings was counterintuitive at first view. The intrapersonal RESP‐RR synchrony was found significantly anti‐phasic, pointing to an anatomical local coordination of inhaling (high RESP values) and reduced respiration rate (low RR values). A highly speculative understanding may follow along these lines: Such synchrony would be consistent with *antara kumbakha* (“inner bated breath”, part of *pranayama*, Indian breath‐control yoga); *antara kumbakha* is a specific exercise, which advises stopping breathing for a moment after having inhaled. In yogic tradition, this technique is instructed in order to relax the mind (Saoji et al. 2018), but to our knowledge there are no findings of its spontaneous occurrence as a sign or consequence of relaxation. A related other speculation concerning the RESP‐RR synchrony finding is that a specific aesthetic response to the music may be involved here: listeners may respond to exciting or suspenseful music passages with bated breath, by momentarily slowing down breathing represented by a phasic reduction of breathing rate (RR low). Such behavior may occur more likely and be more comfortable during the inhalation period of the respiratory cycle (RESP is high), which might also have supported the anti‐phasic intrapersonal RESP‐RR synchrony we found.

A further unexpected finding concerned the EDA‐HRV synchronies, which were significant but in‐phase in both the intra‐ and interpersonal conditions. Their independent presence in both conditions, within and between persons, suggests a reliable finding. Since higher EDA is an exclusively sympathetic signal and higher HRV predominantly a parasympathetic signal, we expected as per RQ1 that they should act antagonistically and thus produce rather anti‐phasic synchronies. This was not the case in the present dataset. Upon closer deliberation, however, there are contexts in which sympathetic and parasympathetic activation may arise conjointly, such as in flow experiences (Csikszentmihalyi 1990) and in positively experienced stress (*eustress*: Lazarus et al. 1980). In flow states, such sympathetic‐parasympathetic co‐activation (Peifer and Tan 2021) was found, where both branches of the Autonomic Nervous System are (moderately) activated, which can entail in‐phase EDA‐HRV synchronies. de Manzano et al. (2010) described that kind of co‐activation as *relaxed alertness* based on their findings in piano players. We speculate that this might also be true in a concert setting, where listeners are seated safely and comfortably and, at the same time, are aroused by aesthetic experiences. Sympathetic‐parasympathetic co‐activation is likewise consistent with the notion of a two‐dimensional autonomic space (Berntson et al. 1993) rather than a one‐dimensional continuum ranging from parasympathetic to sympathetic activation. Both the intrapersonal RESP‐RR synchrony and the intra‐ and interpersonal EDA‐HRV synchronies were unexpected results of our exploratory approach. We have discussed them post hoc by speculative interpretations, which may serve as tentative hypotheses in future research.

To date, research on interpersonal synchrony has predominantly considered intramodal synchronies, and until recently (Tschacher et al. 2025) has largely disregarded leading‐following aspects within the respective synchrony measures. With the present analyses we have extended the range of phenomena considerably by shifting the focus to crossmodal synchronies and to the temporal leading‐following dynamics of variables contributing to such crossmodality. In real life, bodily coordination is unlikely to occur only within the same physiological modality. Shared experiences may shape several physiological systems at once, and these systems may align either simultaneously or with slight temporal offsets. By considering crossmodal synchronies and leading‐following aspects, the present approach moves closer to how physiological coordination likely unfolds in natural situations, and consequently provides a richer picture of how shared experiences become embodied. It also provides insight to answer questions such as: Are different bodily systems coordinated in patterned ways, not just within a person but also across people? Does synchrony occur all at once, or is there a tendency for one bodily process to lead and another to follow?

The present data represents synchronies induced by external stimuli. A more prominent application, however, concerns interpersonal synchrony as a hallmark of social interaction, where synchrony findings have obtained psychological, linguistic, and developmental relevance. With the technology‐driven availability of wearable devices increasing, multivariate time series of various physiological modalities, including body movement, will become the new standard. Therefore, crossmodal synchronies will extend the scope of measures available for social‐interaction studies considerably. At the same time, crossmodal intrapersonal findings open up a largely novel methodology for psychophysiology research in general. Although the synchrony patterns observed in the present study cannot be assumed to generalize directly to interaction contexts, where speech, mutual responsiveness, and reciprocal coordination are likely to shape synchrony, they call for analogous research in social‐interaction studies. It is a methodological benefit that differently caused phenomena—social interaction synchrony and externally induced synchrony—can be studied using the same standardized method.

### 4.1 Benefits and Limitations

A benefit of the present project is the high sample size of up to 703 participants, who provided the physiological time series. A further asset is the ecological validity of the study design: Participants were attending a public live concert, and their primary motivation to attend was listening to the musical presentations, not participating in a physiological experiment. This shared motivation, thus a positive selection of people intrinsically interested in the staged music, may have supported their synchronous responsivity to the stimuli, which likely exceeds the physiological responses that would be captured in a laboratory experiment. Because of its large sample, multiple physiological modalities, and the joint examination of intra‐ and interpersonal synchrony in a naturalistic setting, the present study may provide a useful reference point for future research on physiological synchrony induced by salient stimuli or by the affordances of social interaction. In the past, we implemented the same cross‐correlational methodology in different contexts, studying synchrony *induced* by stimuli in concerts as well as synchrony *arising spontaneously* in social interactions. This has the benefit of methodological standardization, which is a pressing issue in the field of synchrony research (Altmann et al. 2022).

Limitations of the study concern the physiological and technical heterogeneity of the signals and the preprocessing steps that were necessary. Synchrony could only be computed between time series with identical sampling rates, yet the five modalities differ not only in their original form of measurement but may also differ with respect to their temporal characteristics. Accordingly, the slower and more strongly derived variables (especially respiration‐rate and heart‐rate variability) required different preprocessing than the faster, more continuous signals (such as phasic electrodermal activity and respiration behavior). In particular, some measures were obtained more directly from the recorded signal, whereas others were derived from intervals between adjacent peaks using moving windows. As a result, part of the observed synchrony may reflect not only physiological coupling but also differences in signal construction, temporal smoothing, and effective temporal resolution. Consequently, a more cautious interpretation is that the anti‐phasic RESP‐RR synchrony may contain both methodological and physiological factors. Because RR was derived from RESP peak intervals and transformed into a 1‐Hz time series, some coupling may be due to the dependence between the two measures and their preprocessing. In addition, EDA and HRV were derived at different temporal scales in the present study. EDA was recorded as a continuous signal whereas HRV was estimated from overlapping time windows and assigned a value each second; again, the observed coupling may reflect signal construction and temporal smoothing in addition to a specific physiological co‐activation process.

## Author Contributions

**Wolfgang Tschacher:** conceptualization, methodology, software, data curation, formal analysis, writing – original draft, writing – review and editing, funding acquisition. **Anu Tourunen:** conceptualization, supervision, writing – original draft, investigation. **Petra Nyman‐Salonen:** conceptualization, writing – original draft, investigation, supervision. **Virpi‐Liisa Kykyri:** conceptualization, writing – original draft, investigation, supervision.

## Funding

The first author received funding for data collection this article is based on. Funding was received from Volkswagen Foundation for the Experimental Concert Research (ECR) project, No. 93 263.

## Ethics Statement

The procedure was approved by the ethics committee of the Max‐Planck Society (#2702_12), and all research was performed in accordance with relevant guidelines and regulations and with the Declaration of Helsinki.

## Conflicts of Interest

The authors declare no conflicts of interest.

## Data Availability

The data that support the findings of this study are available on request from the corresponding author. The data are not publicly available due to privacy or ethical restrictions.

## Endnotes

^1^

https://experimental‐concert‐research.org/?lang=en.
